# Preventive and social cost implications of Ebola Virus Disease (EVD) outbreak on selected organizations in Lagos state, Nigeria

**DOI:** 10.11694/pamj.supp.2015.22.1.6673

**Published:** 2015-10-11

**Authors:** Babasola Oluseyi Olugasa, Oluwafunmilola Yemisi Oshinowo, Eugene Amienwanlen Odigie

**Affiliations:** 1 Centre for Control and Prevention of Zoonoses, University of Ibadan, Ibadan, Nigeria; 2 Department of Veterinary Public Health and Preventive Medicine, University of Ibadan, Ibadan, Nigeria; 3 Disaster Risk Management Programme, Department of Geography, Faculty of the Social Sciences, University of Ibadan, Ibadan, Nigeria

**Keywords:** Ebola virus disease, hospital, hotel, school, preventive cost, social cost

## Abstract

**Introduction:**

As Ebola virus disease (EVD) continues to pose public health challenge in West Africa, with attending fears and socio-economic implications in the current epidemic challenges. It is compelling to estimate the social and preventive costs of EVD containment in a Nigerian city. Hence, this study was to determine the social and preventive cost implications of EVD among selected public institutions in Lagos, Nigeria, from July to December, 2014.

**Methods:**

Questionnaires and key-informants interview were administered to respondents and administrators of selected hospitals, hotels and schools in Eti-Osa Local Government Area of Lagos State. Knowledge of disease transmission, mortality and protocols for prevention, including cost of specific preventive measures adopted against EVD were elicited from respondents. Descriptive statistics and categorical analysis were used to summarize and estimate social and preventive costs incurred by respective institutions.

**Results:**

An estimated five million, nineteen thousand, three hundred and seventy-nine Naira and eighty kobo (N5,019,379.80) only was observed as direct and social cost implication of EVD prevention. This amount translated into a conservative estimate of one billion, twenty-seven million, ninety-four thousand, seven hundred and fifty-six Naira (N1,027,094,756.10) for a total of four thousand schools, two hundred and fifty-three hospitals and one thousand, four hundred and fifty one hotels in Lagos during the period (July 20-November 20, 2014).

**Conclusion:**

The high cost of prevention of EVD within the short time-frame indicated high importance attached to a preventive policy against highly pathogenic zoonotic disease in Nigeria.

## Introduction

Ebola virus disease epidemic (EVDE) of 2014 in West Africa can simply be described as one of the four main types of disasters. It is a public health emergency involving a sudden onset of contagious disease that affects health, disrupts services and businesses, incurring economic and social costs [1, 2–3]. On August 8 2014, the Director-General of the World Health Organization (WHO) declared the Ebola outbreak in West Africa a Public Health Emergency of International Concern (PHEIC) [4]. Although the WHO was notified in March, 2014 of an outbreak of a communicable disease characterized by fever, severe diarrhea, vomiting, and a high fatality rate in Guinea, which was confirmed as Zaire Ebolavirus (EBOV), its containment had been out of reach, with wide geographic spread, weak and apparently overwhelmed health-care systems, and community mistrust and resistance. The presumed first fatality of the outbreak was in December 2013 in Guinea [4, 5–7].

On July 20 a man flew on commercial plane from Monrovia, Liberia to Lagos, Nigeria. That man took ill and was rushed to the First Consultant Medical Center, Obalende, Lagos, where he later died from the disease on July 25. Medical experts generated a list of 898 contacts. This index case spread the disease to some 18 other people that were diagnosed with the disease across two states. Thus, Nigeria recorded 19 (15 in Lagos State and 4 in Rivers State) laboratory confirmed cases of EVD including the index case. The transmission of the virus came to a halt in September 5, as no new cases of the disease were diagnosed as from that day. By September 24, the EVD isolation and treatment wards had no in-patients. Subsequently, the WHO declared Nigeria EVD-free on Monday, October 20 since no new cases were identified in the country from September 5 [1, 4–5]. EVD outbreak affected adversely the resumption of schools; hospitals were extra cautious and even sentimental as to admitting sick patients. Hospitals that admitted or had contact with confirmed or suspected EVD patients were shut down by health agencies. Hotels hurriedly put in place precautionary measures against accommodating apparently healthy persons due to gripping fear and social panic within communities [5, 6–8]. In short, essential social services, productivity, international trade and transport were suddenly interrupted leading to a significant back drop of public and private activities in Nigeria. Adequate follow-up and contact tracings accomplished the effective management of 18,500 potentially exposed persons to find any new cases among a total of 989 identified contacts was valued at a social and preventive cost [9]. Hence, the rapid and collective responses engaged by all sectors of the economy were laudable.

Like any disaster or international emergency, the 2014 EVD outbreak in Lagos, Nigeria interrupted essential social services, productivity, international trade and transportation. It is therefore important to collate and estimate the cost implications of the emergency responses that were put in place by certain public institutions in Lagos state, Nigeria. This study was therefore, designed to estimate the cumulative social and preventive cost of EVD among selected hospitals, hotels and schools in Lagos state that led to the containment of the disease outbreak in the nick of time during July, 20th to November 31st, 2014.

## Methods

### Study design

The study was descriptive cross-sectional in design. In this study, accessible information were sourced from the Nigerian Centre for Disease Control (NCDC) and the Lagos State Ministry of Health on the number of operational hospitals, hotels and schools available during the period of the survey, July to December, 2014.

### Study setting/area

**Figure 1:**
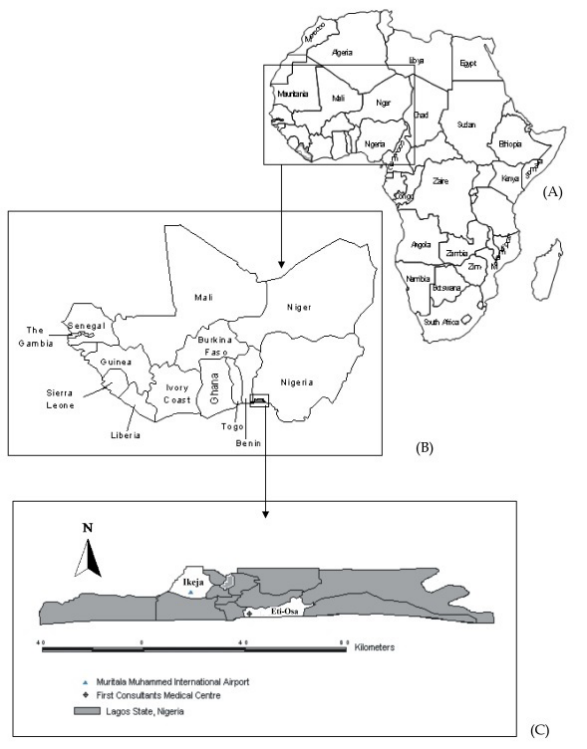
Map of Africa (A), showing West Africa (B) and the study location in Eti-Osa Local Government Area, Lagos State, Nigeria (C)

The study took place in Eti-Osa Local Government Area of Lagos State, Nigeria. Eti-Osa is a high brow metropolitan area with estimated human population of 287,785. It is one of the two local government areas that accommodated the seat of the national capital before the Nigerian capital city moved to Abuja. Within Eti-Osa are several important areas of Lagos, including Victoria Island, and the First Consultant Medical Centre that admitted and attended to a Liberian diplomat that was confirmed the first EVD patient, and index case that brought the disease to Nigeria, via air flight, through Muritala Muhammed International Airport (MMIA). MMIA is in Ikeja Local Government Area, approximately 2 km to Eti-Osa, both within Lagos State (Figure 1). A rich transportation network connects other West African countries to the south-western part of Nigeria, through Lagos. The researchers could not lay hands on the print-out of the number of schools that were registered in Lagos State [10] from the Ministry of Education; personal interview with the proprietors of public and private schools put the figure at 4002 as at September, 2014 [9]. A total of two hundred and fifty-three hospitals and one thousand, four hundred and fifty one hotels were enlisted on the register of public and private organizations operating in Lagos with government approval during the study period [10].

### Sampling technique

A mixed sampling method consisting of a purposively and random sampling technique was used to select respondents at LGA and institution levels. Random sampling by balloting was used to select 14 Schools, 6 hospitals and 5 hotels in Eti-Osa Local Government Area (LGA) of Lagos State, where the index case of EVD in Nigeria was first attended to at the First Consultants Medical Centre, Ikoyi Road, Obalende. Information was elicited from consenting staff of the various institutions that participated in the study. Schools were included in the study because the EVD outbreak led to prolonged delay in school resumption.

### Instruments for data collection, data management and analysis

A validated self-administered semi-structured questionnaire was used to collect primary data for the study. Questionnaires were developed into sections to specifically elicit information on respondents’ socio-demographic characteristics, knowledge on disease transmission and cost implication of the outbreak. Questions that were administered using the key informant interview ranged from preventive to social cost incurred during the time frame. Descriptive statistics, including simple percentage, average, frequency count, tables and charts were used to summarize, analyze and evaluate pertinent variables. Thematic maps were also used to illustrate areas that were notably affected in Lagos State.

## Results

It was observed that majority (92.1%) of the respondents had adequate knowledge of EVD outbreak in Lagos (Table 1). Respondents also showed good understanding of how EVD was transmitted (Table 1), knowledge of its symptoms, and how to prevent the disease, as prescribed by public health authorities. The source of respondents’ knowledge demonstrated was attributed to massive awareness campaign embarked on by the Ministries of Information and National Orientation Agency as well as Non-Governmental Organisations, through the use of electronic and print media.

**Table 1:** Respondent's knowledge of Ebola virus disease outbreak, severity and mode of transmission in Eti-Osa LGA, Lagos state (n = 101)

Statements	N	%
Are you aware of the disease called Ebola Virus Disease (EVD)?		
Yes	101	100.0
EVD is a deadly disease		
Yes	99	98.0
EVD is not a deadly disease		
Yes	3	3.0
EVD can be spread through hand shake		
Yes	80	79.2
EVD can be spread through touching of blood and body fluid of an infected person		
Yes	100	99.0
EVD can be spread through breast feeding by an infected person		
Yes	94	93.1
EVD can be spread through air		
Yes	28	27.7
EVD can be spread through handling of unsterilized needles or medical equipment used for an infected person		
Yes	100	99.0
Eating of bush meat like Bat, Monkey can cause Ebola		
Yes	91	90.1
Eating of Biter Kola can prevent EVD		
Yes	4	4.0
Drinking and washing with salt water can prevent EVD		
Yes	9	92
High temperature is one of the symptoms of EVD	94	93.1
Yes		

Majority of the proprietors of schools, hospitals and hotels committed funds to prevent EVD in their facilities. The use of infrared thermometer for non-contact body temperature check was a common cost item. Similarly, potable hand sanitizers and wash hand basins was a cost item in the institutions (Table 2). Expenses incurred on coordinated awareness campaigns within these institutions included use of posters, flip charts and training of staff on preventive practices. These expenses amounted to approximately two hundred and eight thousand Nigerian naira (N208,000.00) (Table 2).

**Table 2:** Cost estimates of procured EVD prevention materials in Eti-Osa, local government area of Lagos State, Nigeria, July – November, 2014

Organisation	Average Expenses on Hand Sanitizer (N)	Average Expenses on Infrared Thermometer (N)	Average Expenses on Buckets (N)	Average Expenses on Soap (N)	Average Expenses on Awareness Campaign (N)	Average Expenses on Staff Training (N)	Average Expenses on Other related Miscellaneous Materials (N)
**Schools**	18,957.14	19,321.43	5,282.14	2,571.43	37,500.00	26,428.57	16,285.71
**Hospitals**	27,875	41,583.33	9,583.33	2,916.67	81,333.33	87,500.00	32,500.00
**Hotels**	43,396.00	41,600.00	13,160.00	4,000.00	110,000.00	69,000.00	29,000.00
**Total**	**90,228.14**	**102,504.76**	**28,025.47**	**9,488.1**	**228,833.33**	**182,928.57**	**77,785.71**

In all, the costs declared by respondents was approximately five million, nineteen thousand, three hundred and seventy-nine Naira and eighty kobo (N5,019,379.80) only. The mean expenditure on these institutions translated to an approximate one billion, twenty-seven million, ninety-four thousand, seven hundred and fifty-six Naira (N1, 027,094,756.10) on all related institutions (hospitals, hotels and schools) within Lagos state during the study period (Table 3).

**Table 3:** Direct estimation of cost implications of EVD preventive measures among hospitals, hotels and schools in Lagos State, Nigeria, July – November, 2014

Organization	Average institutional Expenditure (N)	No. of institutions in Lagos State	No of Institutions selected for the study	Direct Total Estimate of Costs Incurred (N)	Extrapolated Cost Implications (N)
**Schools**	126,346.40	4,000	14	1,768,849.60	505,385,600.00
**Hospitals**	283,291.70	253	6	1,699,750.20	71,672,800.10
**Hotels**	310,156.00	1,451	5	1,550,780.00	450,036,356.00
**Total**	**719,794.1**	**5,704**	**25**	**5,019,379.80**	**1,027,094,756.10**

## Discussion

In this study, respondents’ knowledge of EVD in Eti-Osa local government area of Lagos state were the index case in Nigeria occur was very high. This high knowledge may probably be due to the high alert and apprehension among residents and the pro-active response of the Federal Government of Nigeria to the potentially devastating disease outbreak. The high cost expended in mitigating the disease among public institutions in Lagos clearly reflects the importance attached to the disease as it lasted within the shortest possible period in Nigeria. This however corroborates the World Health Organization's view of the proactive efforts taking by the Nigerian government in putting a stop to the spread of the disease within the country [4]. The unprecedented loss of human lives and capital from the 2014 EVD outbreaks in West Africa, although effectively contained in Lagos State, Nigeria, can take even a greater toll through the continual and prolonged loss of human lives, disease and emigration. Cost expenditures placed on staff training, awareness and sensitization campaigns in public institutions in Eti-Osa, during the period of outbreak were higher compared to expenses made on the procurement of related EVD prevention materials. This evidently shows the importance given to health education as a primary level of disease control and prevention and the need to re-orientate the public on the values attached to the need to modify risky behavioural practices [11].

A major lesson from this is that there is a need to reinforce disease preparedness and human-animal disease surveillance in Nigeria and indeed the entire West African sub-region [12]. In situations of protracted outbreaks, adverse effect on the national economy could occur. We reckon that the preventive and social cost of EVD on the financial capital of Nigeria, and indeed of the entire country is a critical pointer to the merit of continual vigilance against highly pathogenic zoonotic disease events. Obviously other West African nations critically affected by EVD could not monster the financial muscle shown by Lagos State. A disaster of this magnitude might have negatively affected the national savings, insurance, and access to credit.

It is however possible to learn from these events and develop more positive attitude and best practices towards strengthening disease surveillance practice and education in West Africa. In particular, effective educational adaptation to EVD experience in West Africa requires shifting from response and recovery to awareness, preparedness and accurate prediction skills [13, 14–15]. It is especially critical to integrate relevant aspects of indigenous risk factors into awareness and preparedness against EVD as advocated in other disaster events [11, 12–15]. The EVD outbreak corroborates the stakeholders’ steps to improve postgraduate programmes for surveillance of human-animal disease at the University of Ibadan, Nigeria with the introduction of a Systematic Epizootiology programme and Certificate of Participation in Human-Animal Disease Surveillance at the University of Ibadan [16, 17].

## Conclusion

Although, public knowledge on EVD was high, the social and preventive costs expended on EVD in Lagos State, Nigeria, were massive. Schools could not resume as scheduled because of fear of spreading the virus. Public and large gatherings, including religious programmes were minimized because of fear of EVD. There was an increase in the cost of doing business as a result of the outbreak in Lagos during the study period. The high cost expended is pointer to the need for continual vigilance and investment in surveillance and control at source rather than detection and response.
